# Spinal muscular atrophy within Amish and Mennonite populations: Ancestral haplotypes and natural history

**DOI:** 10.1371/journal.pone.0202104

**Published:** 2018-09-06

**Authors:** Vincent J. Carson, Erik G. Puffenberger, Lauren E. Bowser, Karlla W. Brigatti, Millie Young, Dominika Korulczyk, Ashlin S. Rodrigues, KaLynn K. Loeven, Kevin A. Strauss

**Affiliations:** 1 Clinic for Special Children, Strasburg, Pennsylvania, United States of America; 2 Franklin and Marshall College, Lancaster, Pennsylvania, United States of America; University of Edinburgh, UNITED KINGDOM

## Abstract

We correlate chromosome 5 haplotypes and *SMN2* copy number with disease expression in 42 Mennonite and 14 Amish patients with spinal muscular atrophy (SMA). A single haplotype (A1) with 1 copy of *SMN2* segregated among all Amish patients. *SMN1* deletions segregated on four different Mennonite haplotypes that carried 1 (M1a, M1b, M1c) or 2 (M2) copies of *SMN2*. DNA microsatellite and microarray data revealed structural similarities among A1, M1a, M1b, and M2. Clinical data were parsed according to both *SMN1* genotype and *SMN2* copy number (2 copies, n = 44; 3 copies, n = 9; or 4 copies, n = 3). No infant with 2 copies of *SMN2* sat unassisted. In contrast, all 9 Mennonites with the M1a/M2 genotype (3 copies of *SMN2*) sat during infancy at a median age of 7 months, and 5 (56%) walked and dressed independently at median ages of 18 and 36 months, respectively. All are alive at a median age of 11 (range 2–31) years without ventilatory support. Among 13 Amish and 26 Mennonite patients with 2 copies of *SMN2* who did not receive feeding or ventilatory support, A1/A1 as compared to M1a/M1a genotype was associated with earlier clinical onset (p = 0.0040) and shorter lifespan (median survival 3.9 versus 5.7 months, p = 0.0314). These phenotypic differences were not explained by variation in *SMN1* deletion size or *SMN2* coding sequence, which were conserved across haplotypes. Distinctive features of SMA within Plain communities provide a population-specific framework to study variations of disease expression and the impact of disease-modifying therapies administered early in life.

## Introduction

Biallelic deletions of *SMN1* cause spinal muscular atrophy (SMA; MIM# 253300), a common monogenic cause of infant mortality characterized by progressive degeneration of lower motor neurons [1–4]. *SMN1* encodes survival motor neuron protein (SMN), which is 294 amino acids in length (32 kDa) and interacts with other proteins to influence ribonucleoprotein assembly, ubiquitin homeostasis, cytoskeletal dynamics, endocytosis, and neuromuscular junction stability [5–7]. SMN is expressed in tissues throughout the body but lower motor neurons are particularly vulnerable to its absence [4, 6].

The SMA locus on human chromosome 5q13 contains telomeric *SMN1* and centromeric *SMN2*, both of which produce SMN protein, and 8 other genes aligned within a 2 Mb vicinity (*OCLN*, *GTF2HCDC*, *SERF1B*, *SERF1A*, *NAIP*, *GTF2H2*, *LOC647859*, and *LINC02197*). This region is subject to deletions, duplications, and rearrangements that damage *SMN1* and its neighboring genes and also give rise to a variable number of *SMN2* copies [7]. Compared to *SMN1*, *SMN2* contains a base difference (c.850C>T) that excludes exon 7 from approximately 90% of mRNA transcripts to produce a truncated protein (SMNΔ7; 282 amino acids, 30.5 kDa) that is non-functional and rapidly degraded [8]. Residual intact SMN translated from each *SMN2* copy partially compensates for *SMN1* deficiency such that genomic *SMN2* copy number correlates inversely with disease severity [9–11]: two *SMN2* copies commonly segregate with a severe (type 1) SMA phenotype whereas three or more copies correlate with later disease onset and slower progression [12, 13].

Within ethnically mixed (i.e. outbred) populations, carrier frequencies for pathogenic *SMN1* deletions are between 1.4% (African American) and 2.1% (Caucasian)[14, 15], but range from 3 to 13% in certain endogamous populations of Israel [16, 17], Hungary [18], Spain [19], France [20], and the United States [21]. In contrast to the individuals who comprise a multiethnic cohort, patients from a common founder lineage are typically homozygous for the same *SMN1* mutation, share a large proportion of background genetic material, experience similar dietary and environmental exposures, live in comparable socioeconomic conditions, and adhere to similar patterns of medical resource utilization. Thus, founder groups naturally control for many genetic and environmental variables that complicate natural history studies in outbred cohorts [22, 23].

Spinal muscular atrophy is relatively common among Old Order Amish and Mennonite (Plain) populations. Contemporary Plain people descended from a small group of Swiss Anabaptist founders who migrated to the New World during the early 18^th^ century and have remained isolated in small demes across North and South America [24]. Here, we investigate ancestral chromosome 5 haplotypes in relation to SMA phenotype among 42 Mennonite and 14 Amish individuals harboring 2 (n = 44), 3 (n = 9), or 4 (n = 3) copies of *SMN2*. This is the first comprehensive clinical description of SMA within Plain communities and provides a unique lens through which to view the natural history of disease undistorted by technological interventions, genetic variability, and heterogenous approaches to care.

## Results

### Genotype

#### SMA haplotype structure and segregation

**Fig 1:**
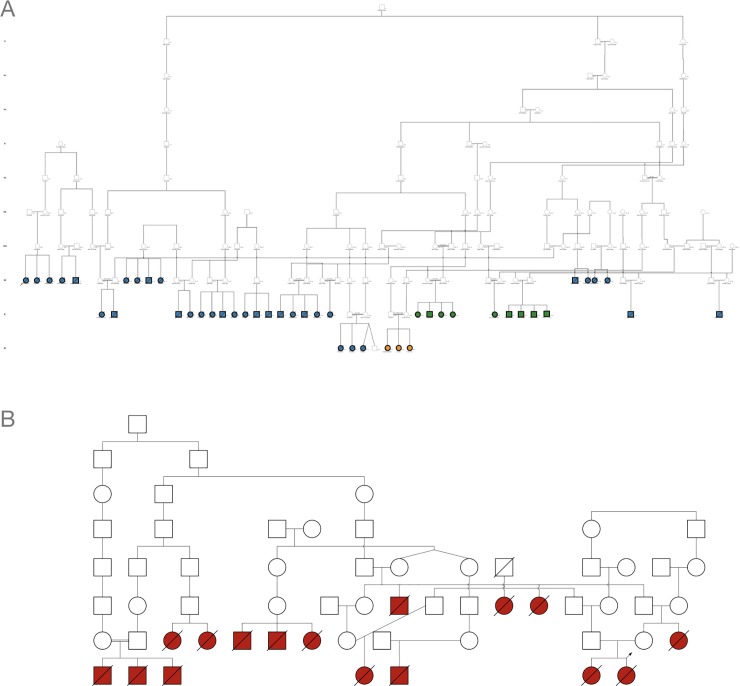
Spinal muscular atrophy in Mennonite and Amish pedigrees. (A) Forty-two Mennonite SMA patients with homozygous deletions of *SMN1* traced to a common founder through 11 generations but had variable numbers of *SMN2* copies (blue, 2 copies of *SMN2;* green, 3 copies of *SMN2;* orange, 4 copies of *SMN2*). (B) Although we could not connect 14 Amish SMA patients using extant genealogical records, all were homozygous for the same SMA haplotype (red, 2 copies of *SMN2*) indicating common ancestry.

**Fig 2:**
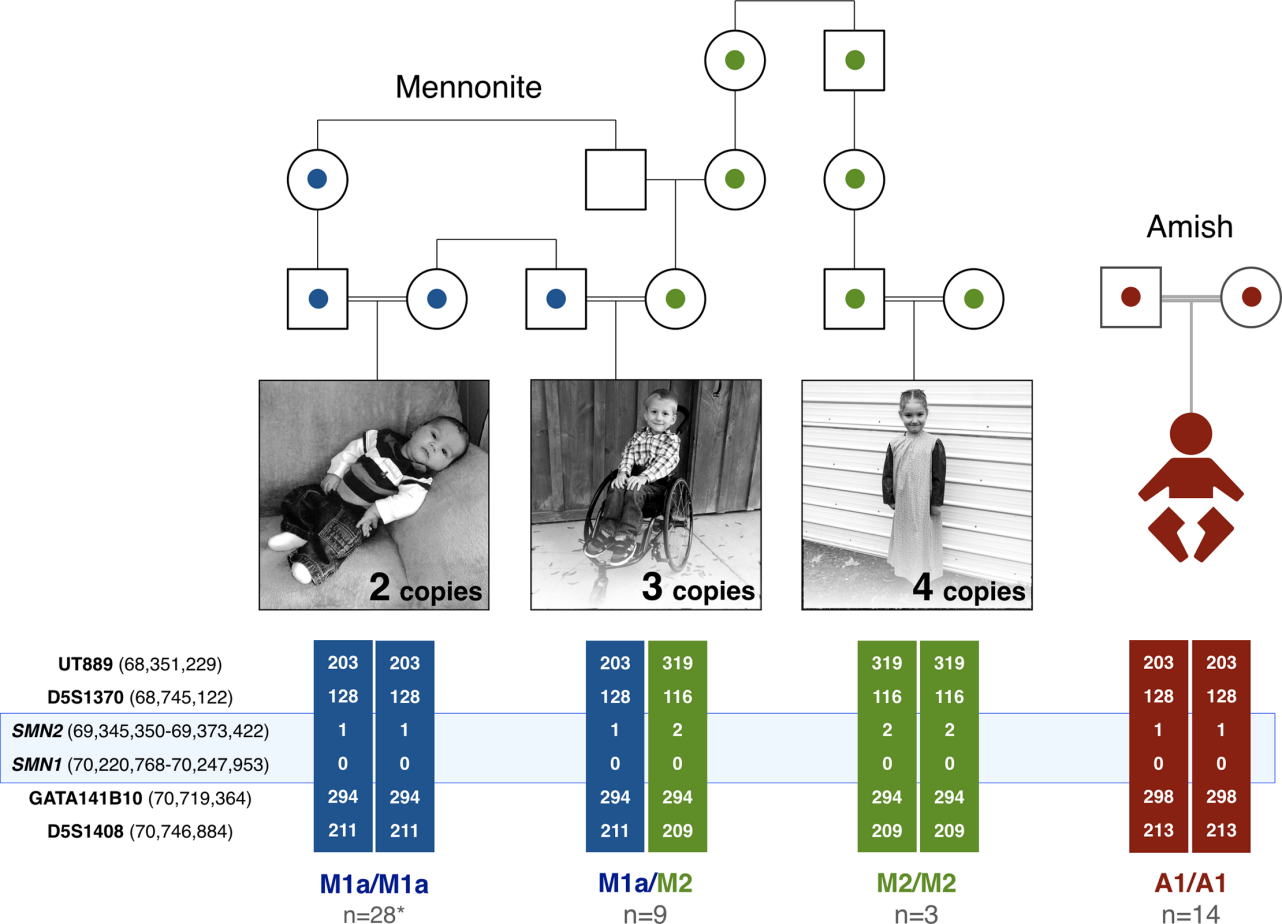
Segregation of SMA haplotypes. (A) To map SMA haplotypes, we used DNA microsatellite markers in close proximity to *SMN1* and *SMN2* on chromosome 5q13. Among Mennonites, pathogenic *SMN1* deletions segregated on four different haplotypes that harbored either 1 (blue; M1a, M1b, M1c) or 2 (green; M2) copies of *SMN2* to produce genotypes comprising 2 (M1/M1; n = 30), 3 (M1/M2; n = 9), or 4 (M2/M2; n = 3) copies of *SMN2*. (B) A single ancestral haplotype (A1) with 1 copy of *SMN2* was inferentially shared among all 14 Amish patients homozygous for the A1/A1 genotype (red; 2 copies of *SMN2*).

We used DNA microsatellites and 2.6 million-marker single nucleotide polymorphism (SNP) microarrays to study the 5q13 SMA locus of Mennonite SMA patients and their extant family members, all of whom traced back 11 generations to a common ancestor (Fig 1A). A pathogenic *SMN1* deletion segregated on two major Mennonite haplotypes (M1a, M2) that encompassed 1 and 2 copies of *SMN2*, respectively (Fig 2). M1a/M1a (2 copies *SMN2*, n = 28), M1a/M2 (3 copies *SMN2*, n = 9), and M2/M2 (4 copies *SMN2*, n = 3) were the predominant SMA genotypes, segregating predictably with severe (70%), moderate (20%), or mild (10%) disease (Fig 2).

**Fig 3:**
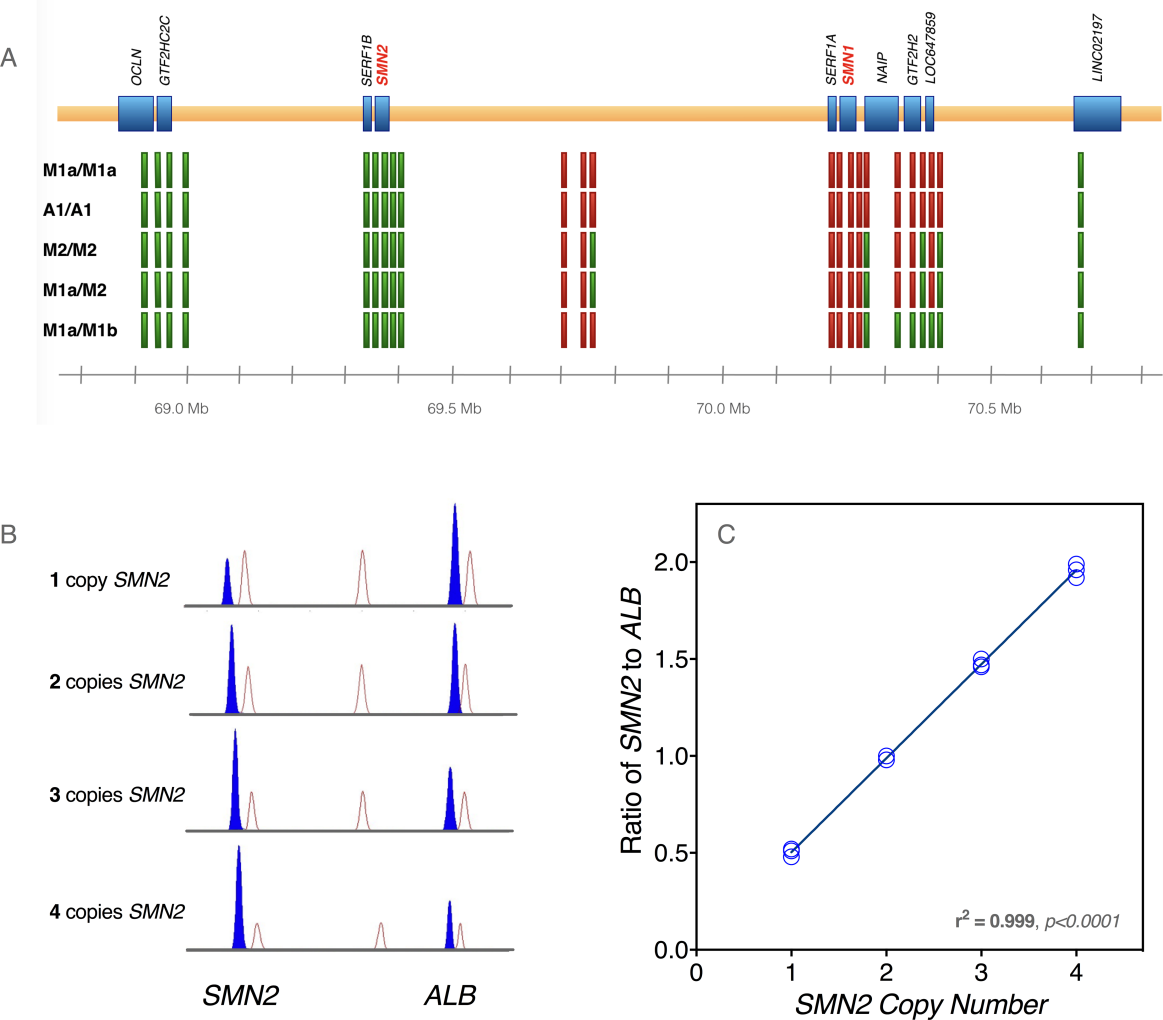
Characterization of SMA haplotypes. (A) To characterize the extent of *SMN1* deletions, we performed PCR on loci that mapped to multiple sites in the region. By selecting amplicons which exhibited sequence changes between loci, we were able to assess the presence or absence of each locus after PCR amplification and Sanger sequencing. Green blocks represent loci that were present, and red blocks denote deleted loci. The samples (i.e. haplotype combinations) are listed on the left side of the figure. (B) Competitive PCR was used to calculate *SMN2* copy number in our patient cohort. Samples were subjected to multiplex PCR with limiting deoxynucleotide triphosphates and the resulting amplicons were size-fractionated on an ABI 3130 Genetic Analyzer. Samples with greater *SMN2* copy number demonstrated increased generation of *SMN2*-specific product versus an internal control locus (albumin gene, *ALB*). (C) The area under the curve for each amplicon, as provided by the Sequencing Analysis software, was used to calculate the ratio of *SMN2*-specific product to *ALB* product. For *SMN2* copy number from 1–4, three separate samples were PCR amplified and analyzed, and the *SMN2/ALB* ratios were highly correlated with *SMN2* copy number.

Two minor Mennonite SMA haplotypes (M1b and M1c) each contained 1 copy of *SMN2* and were compound heterozygous with M1a in a single patient. M1b did not share proximal microsatellite markers with M1a but matched distally (S1 Table, Sample 21358), indicating it might originate from a rare recombination event on M1a. However, deletion mapping revealed M1a and M1b to be altogether different (Fig 3A). Although no patient sample was available, we inferred a potential second minor Mennonite haplotype (M1c) from the mother of an affected child. She carried an *SMN1* deletion and 1 copy of *SMN2* but microsatellite markers suggested that she carried a rare double recombinant M1a or a novel haplotype (S1A Fig, Individual 4273). Hereafter, and as indicated in Tables and Figures, the simplified designation ‘M1’ refers to any one of three Mennonite genotypes (i.e. M1a/M1a, M1a/M1b, or M1a/M1c) that confers 2 copies of *SMN2*.

A single SMA haplotype (A1) containing 1 copy of *SMN2* segregated in Old Order Amish families (Figs 1B and 2). Although we had DNA samples from only two affected children, microsatellite and *SMN1* copy number data from additional sibships confirmed segregation of A1 among all 14 Amish patients (Fig 1B and S1 Fig).

#### Deletion mapping

A1 and M1a haplotypes harbored similar deletions that encompassed all functional transcripts of *SMN1*, *SERF1A*, *NAIP*, and *GTF2H2* (Fig 3A). They had remarkably similar SNPs and shared identical proximal microsatellite alleles (S2 Table). Although microsatellite markers differed distally, A1 and M1a showed considerable allele sharing on either side of *SMN1* and shared telomeric SNPs with the minor M2 haplotype. Taken together, these data might be explained by (1) a common but ancient origin for all 3 haplotypes which allowed for microsatellite markers to mutate to new alleles over time; (2) independent mutational events that occurred on a permissive (susceptibility) allele, as has been suggested for certain haplotypes associated with Fragile X syndrome [25–27]; or 3) a chance event attributable to a high frequency of the underlying haplotype in the parental population.

#### SMN2 sequencing and copy number

We performed complete *SMN2* sequencing of Amish and Mennonite samples with homozygous A1/A1 and M1a/M1a genotypes, respectively. Primers were designed to amplify all 9 exons of SMN2 as well as intron-exon boundaries and near-intronic regions. *SMN2* sequences from A1 and M1a haplotypes were identical. Competitive PCR allowed us to infer *SMN2* copy number based on its relationship to copies of the albumin gene (*ALB*; Fig 3B).

#### SMA carrier frequency

To estimate SMA carrier frequency among Old Order Mennonites, we interrogated whole exome sequencing (WES) data from 735 Mennonite control samples using the CLAMMS algorithm to detect DNA copy number variations from WES read depth [28]. This allowed us to identify 42 Mennonite controls with deletions of both *GTF2H2* and *NAIP*. These samples were then tested using competitive PCR to confirm co-deletion of *SMN1* in 27 individuals. We thus arrived at an empirical SMA carrier frequency of 3.7% (27 of 735) and minor allele frequency of 1.8% among North American Mennonites. Old Order Amish control data were insufficient to reliably calculate an SMA carrier frequency.

### Phenotype

#### Diagnosis

**Fig 4:**
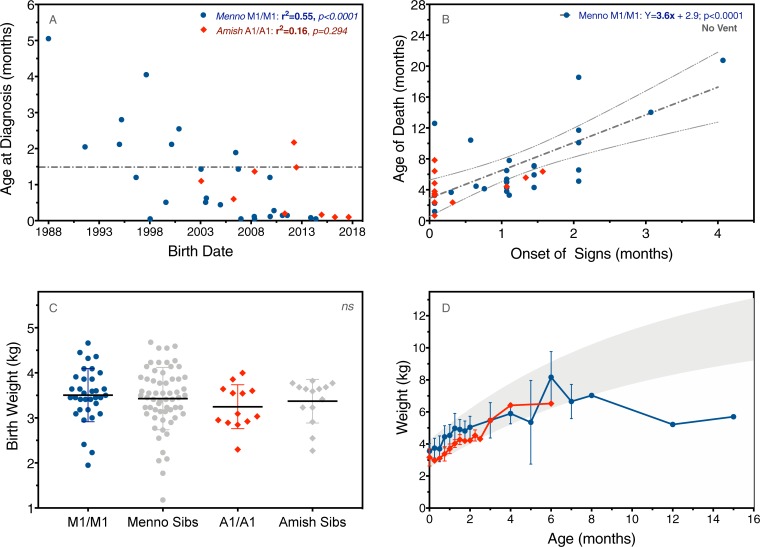
Diagnosis, prognosis, and growth in patients with 2 copies of *SMN2*. (A) Age of diagnosis correlated to birth date (i.e. children born more recently were diagnosed earlier; r_s_ = -0.71, p<0.0001). Among A1/A1 (red diamonds; n = 7) and M1/M1 (blue circles; n = 9) homozygotes born within the last decade, 15 (94%) were diagnosed before 6 weeks of age (gray dashed line). (B) There was a strong linear correlation between clinical onset and age of death among Mennonite but not Amish patients with 2 *SMN2* copies (gray dashed line with 95% confidence bands; p<0.0001). (C) M1/M1 (blue circles) and A1/A1 (red diamonds) homozygotes and their unaffected siblings (gray symbols) had similar birth weights. (D) Amish and Mennonite children with 2 copies of *SMN2* remained on the growth curve, without gastrostomy, until about 6 months of age (gray shaded area represents World Health Organization reference data, 5^th^ to 95^th^ percentile).

Tables 1 and 2 summarize herald signs, motor milestones, and survival according to genotype and *SMN2* copy number. Median age of diagnosis was 0.1 months for Amish infants (A1/A1) with 2 copies of *SMN2* and 1.1, 8.5, and 30 months, respectively, for Mennonite children with 2 (M1/M1), 3 (M1a/M2), or 4 (M2/M2) copies (Table 1). Age of diagnosis correlated inversely with birth date among Mennonite (but not Amish) children with 2 *SMN2* copies (r = -0.74, p<0.0001)(Fig 4A). Molecular diagnosis preceded clinical onset for 24 (43%) of 56 patients in our cohort. Among A1/A1 (n = 7) and M1a/M1a (n = 9) homozygotes born within the last decade, 15 (94%) were diagnosed before 6 weeks of age (median age 3 days)(Fig 4A).

**Table 1:** Presenting signs and gross motor development of Amish (n = 14) and Mennonite (n = 42) children with spinal muscular atrophy (SMA) according to haplotype.

		Amish SMA		Mennonite SMA						
		A1/A1, 2 copies *SMN2* (n = 14)		M1/M1, 2 copies *SMN2* (n = 30)*		M1a/M2, 3 copies *SMN2* (n = 9)		M2/M2, 4 copies *SMN2* (n = 3)		
		Percent	Median (range)	Percent	Median (range)	Percent	Median (range)	Percent	Median (range)	P value^†^
**Age of Diagnosis (months)**			0.1 (0–2.1)		1.1 (0–5)		8.5 (0–30)		30 (8–51)	
**Presenting Signs**										
	Low muscle tone or power	100%		97%		-		100%		>0.999
	Decreased fetal movement	71%		20%		-		-		***0*.*0019***
	Weak cry	71%		17%		-		-		***0*.*0007***
**Motor Milestones (months)**^¶^										
	Roll back to front	-		20%	1.4 (0.7–4)	78%	4 (2.5–5)	100%	4 (4–5)	0.1547
	Sit independently	-		-		100%	7 (6–8)	100%	5.5 (5.4–6)	na
	Crawl on all fours	-		-		75%	9 (7–10)	100%	6.8 (6.8–7)	na
	Walk independently	-		-		56%	18 (12–42)	100%	10	na
** **	Run	-		-		11%	16	100%	15	na

*Includes 28 children with the major homozygous M1a/M1a haplotype as well as 2 compound heterozygotes for M1a/M1b and M1a/M1c, which also confer 2 copies of *SMN2*.

^†^Fisher’s exact test comparing Mennonite (haplotype M1/M1, n = 30) to Amish (haplotype A1/A1, n = 14) SMA patients with 2 copies of *SMN2*.

^¶^Percent calculated for children old enough to achieve requisite skill.

*Abbreviations*: A1, Amish SMA haplotype with 1 copy of *SMN2*; M1, Mennonite SMA haplotype with 1 copy of *SMN2*; M2, Mennonite SMA haplotype with 2 copies of *SMN2*; na, not applicable; SMA, spinal muscular atrophy.

**Table 2:** A1/A1 (n = 14) and M1/M1 (n = 30)* infants with 2 copies of *SMN2*.

** **		Mean (SD)	Median (range)	P value^†^
**Birth Weight (kg)**				0.1470
	*Amish* A1/A1	3.1 (0.5)	3.0 (2.3–3.6)	
** **	*Mennonite* M1/M1	3.5 (0.6)	3.5 (2.0–4.7)	
**Onset of Signs (months)**				***0*.*0040***
	*Amish* A1/A1	0.3 (0.6)	0 (0–1.5)	
** **	*Mennonite* M1/M1	1.1 (0.9)	1.0 (0–4.0)	
**Age of Death (months)**^¶^				***0*.*0314***
	*Amish* A1/A1	3.9 (2.1)	3.9 (0.7–6.5)	
** **	*Mennonite* M1/M1	7.3 (4.9)	5.7 (1.2–20.8)	

*M1/M1 Mennonites include those with M1a/M1a (n = 28), M1a/M1b (n = 1), and M1a/M1c (n = 1) haplotypes; all harbor two copies of *SMN2*.

^¶^Age of death statistics only include the subgroup of Amish (A1/A1, n = 13) and Mennonite (M1/M1, n = 26) children who did not receive mechanical ventilatory support.

^†^Fisher’s exact test comparing children with 2 copies of *SMN2* from either the Amish (A1/A1) or Mennonite (M1/M1) haplotype combinations.

*Abbreviations*: SD, standard deviation; SMA, spinal muscular atrophy.

Clinical onset differed by *SMN2* copy number and 5q13 genotype (Tables 1 and 2). Weakness was common to all SMA types and noted earliest in children with the A1/A1 genotype (p = 0.0040)(Table 2), which was more commonly associated with reduced fetal movement (71%; p = 0.0019) and a weak perinatal cry (71%; p = 0.0007)(Table 1). Among Mennonite but not Amish patients with 2 *SMN2* copies, there was a strong correlation between clinical onset and age of death (r = 0.70, p<0.0001)(Fig 4B). Birth weight was similar for M1/M1 and A1/A1 homozygotes as well as their unaffected siblings (Fig 4C), and both Amish and Mennonite children with 2 copies of *SMN2* remained on the growth curve, without gastrostomy, until about 6 months of age (Fig 4D).

#### Motor development

**Fig 5:**
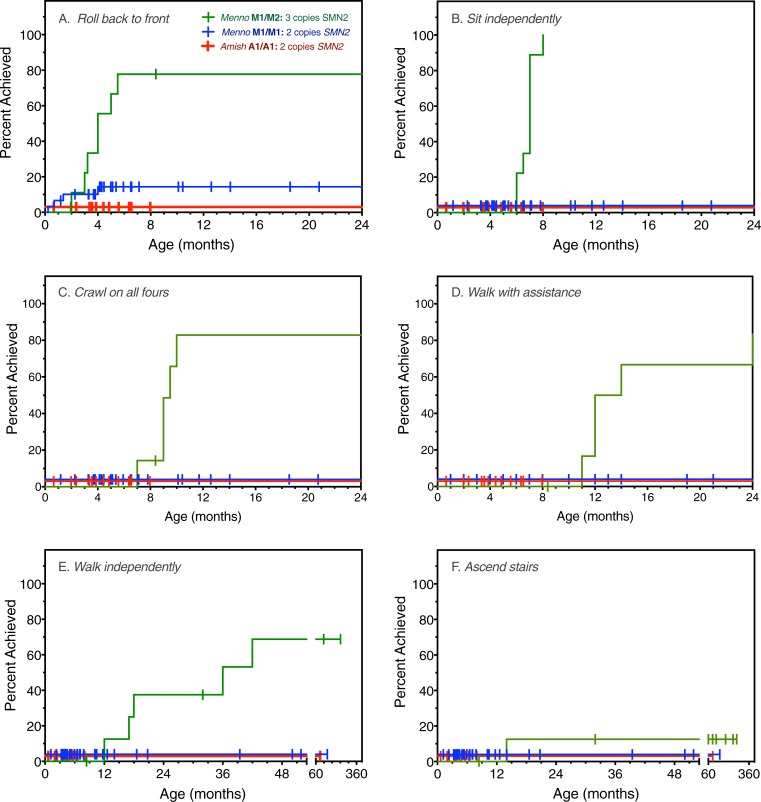
Motor milestone acquisition and *SMN2* copy number. Time to motor milestone acquisition is plotted for Mennonite (blue; M1/M1, n = 30) and Amish (red; A1/A1, n = 14) patients harboring 2 copies of *SMN2* as compared to Mennonites with 3 copies of *SMN2* (green; M1/M2, n = 9). Perpendicular symbols indicate subjects unable to achieve a skill at the time of census (or death). Milestones depicted include rolling back to front (A), independent sitting (B), crawling on all fours (C), walking with assistance (D), independent walking (E), and ascending stairs (F). One child with 3 copies of *SMN2* walked and ascended stairs at 12 and 14 months of age, respectively. Note the divided X-axis in panels E and F.

Among 30 Mennonite (M1/M1) and 14 Amish (A1/A1) infants with 2 copies of *SMN2*, only 6 (all with the M1a/M1a haplotype) rolled back to front; none did so after age 4 months or attained additional motor skills (Table 1 and Fig 5). All 9 individuals with the M1a/M2 genotype (3 copies of *SMN2*) sat unassisted during infancy at median age 7 months and 5 (56%) walked and dressed independently at median ages of 18 and 36 months, respectively. One M1a/M2 patient even learned to ascend stairs, and did so at the remarkably young age of 14 months (Fig 5). All 3 SMA children with 4 *SMN2* copies (M2/M2) achieved motor skills as appropriate for census ages of 0.5, 2.7, and 6.3 years (Table 1).

#### Morbidity

Dysphagia (90%) and pulmonary morbidity predominated among children with 2 copies of *SMN2*. Individuals with 3 *SMN2* copies suffered primarily from musculoskeletal complications, including joint contractures (44%) and scoliosis (56%), in 3 cases culminating in surgical spine fusion. Among children with 2 copies of *SMN2*, only 5 (11%; 1 Amish, 4 Mennonite) were fed by gastrostomy and received daily bilevel positive airway pressure (BiPAP). No patient with the M1a/M2 (3 copies *SMN2*) or M2/M2 (4 copies *SMN2*) genotype required gastrostomy or ventilatory support.

#### Mortality

**Fig 6:**
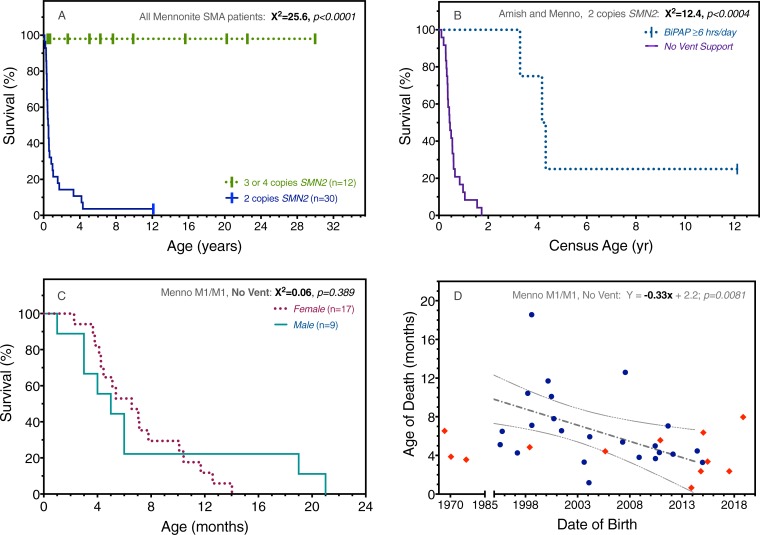
Survival in relation to *SMN2* copy number, ventilatory support, sex, and birth date. (A) All SMA patients with 3 or 4 copies of *SMN2* (green) are presently living, whereas 29 (97%) of 30 with 2 *SMN2* copies (blue) died at a median age of 6.2 months. (B) Among a total of 44 patients with 2 copies of *SMN2* (M1/M1, n = 30; A1/A1, n = 14), bilevel positive airway pressure (BiPAP) for ≥6 hours daily (n = 5) increased median survival from 5.3 to 51 months (p<0.0004). (C) When controlled for BiPAP use and genotype (e.g. M1/M1, no BiPAP, as depicted in Panel C), there was no survival difference based on sex. (D) Paradoxically, date of birth and age of death were inversely correlated for Mennonites with the M1/M1 genotype (Pearson r = -0.51, p = 0.0081).

All patients with 3 (M1a/M2) or 4 (M2/M2) copies of *SMN2* are alive at a median age of 7.9 (range 1.3–30.9) years (Fig 6A). Overall median survival for children with 2 copies of *SMN2* was 6.2 months, but this was strongly influenced by regular use of BiPAP. For 39 children with 2 copies of *SMN2* who did not receive routine ventilatory support, median survival was 5.3 (range 0.5–21) months, whereas 5 patients treated with BiPAP survived to a median age of 51 (range 39–92) months (chi-squared 12.4, p = 0.0004)(Fig 6B). When controlled for BiPAP use and genotype, there was no survival difference based on sex (Fig 6C).

In contrast to historical trends [29], there was no correlation between date of birth and age of death among Amish (A1/A1) patients. Paradoxically, these were inversely correlated (Pearson r = -0.51, p = 0.0081) for Mennonites with the M1/M1 genotype (Fig 6D). This might reflect patterns of palliative care that have emerged in response to the community’s collective experience with type 1 SMA (as discussed below).

**Fig 7:**
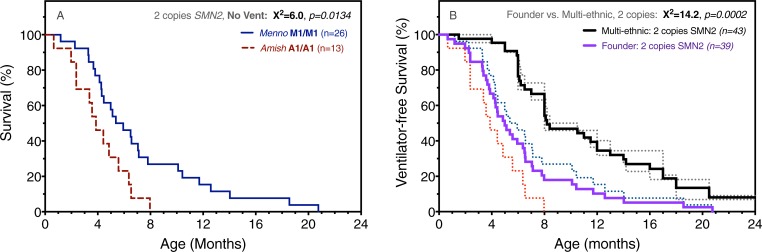
Survival comparisons among Amish, Mennonite, and outbred cohorts. (A) For infants who received no gastrostomy or ventilatory support, median survival of A1/A1 (dashed red; n = 13) and M1/M1 (solid blue; n = 26) homozygotes was 3.9 and 5.7 months, respectively (chi-squared 6.0, p = 0.0143). (B) We compared aggregate (solid purple) survival data for A1/A1 and M1/M1 ‘founder’ cohorts to ventilator-free survival from two ‘multi-ethnic’ outbred cohorts (References 29 and 10) that in aggregate (solid black line) encompass 43 SMA infants with 2 copies of *SMN2*. When permanent ventilatory support (i.e. ≥16 hours daily) is taken as a proxy for death, Amish and Mennonite founder populations had shorter median survival (5.0 versus 8.2 months; chi-squared 14.2, p = 0.0002).

Despite similar *SMN1* deletion structure and *SMN2* copy number, M1/M1 and A1/A1 genotypes were associated with differential survival (Fig 7A). In the absence of gastrostomy or ventilatory support, median survival for A1/A1 (n = 13) and M1/M1 (n = 26) homozygotes was 3.9 and 5.7 months, respectively (chi-squared 6.0, p = 0.0143).

Finally, we compared aggregate A1/A1 and M1/M1 data to ventilator-free survival from two carefully conducted prospective natural history studies published in 2014 (Pediatric Clinical Research [PNCR] Network)[30] and 2017 (NeuroNEXT initiative)[10]. Both defined permanent ventilatory support as a proxy for death and together encompassed 43 SMA infants with 2 copies of *SMN2*. Compared to heterogenous cohorts with access to modern medical care, Amish (A1/A1) and Mennonite (M1/M1) patients had shorter median survival (5.0 versus 8.2 months; chi-squared 14.2, p = 0.0002)(Fig 7B).

## Discussion

### SMA in founder populations

Contemporary Plain people descended from just a few hundred Swiss-German Anabaptists and now comprise more than 800,000 individuals living in isolated demes throughout North and South America [31]. Transatlantic migrations during the 18^th^ century reduced genetic diversity within founding settlements and set the stage for genetic drift; over successive generations, some rare alleles became common while others became extinct [24]. Driven by these mechanisms, the Mennonite SMA carrier frequency equilibrated at its present value of 3.7%, which extrapolates to a disease incidence of 1 per 2,800 newborns. Population control data are insufficient to measure carrier frequency among the Amish, but diagnostic rates in our laboratory over the last decade suggest it approximates 3% (Fig 4A).

A high incidence of SMA is not unique to Plain populations. In 1977, Fried and Mundel estimated an SMA carrier frequency of 10% among Egyptian Karaites, a group socially and reproductively isolated for more than 10 centuries. Subsequently, carrier frequencies between 3 and 13% were identified within founder populations worldwide [16–21]. Among these are the Hutterite people, an Anabaptist group descended from 16^th^ century South German founders who migrated east from Austria-Tyrol to settle in Canada, Montana, and the Dakotas [31]. Their unique ancestral roots are reflected in a single extant SMA haplotype that contains 2 copies of *SMN2* and segregates with a carrier frequency of 12.5% in colonies of South Dakota [21].

### Genotype-phenotype correlations

Jordanova and colleagues first explored the relationship between ancestral haplotypes and SMA phenotype among 32 Romani families living throughout Hungary and Bulgaria [18]. Using 5 microsatellite markers, they delineated three dominant haplotypes (A, B, and C) which segregated in four combinations (A/A, A/B, A/C, and C/C) to cause profoundly different disease patterns. They postulated that rearrangements on a single ancestral chromosome produced haplotypes A, B, and C, and attributed clinical differences to resulting variations of *SMN2* copy number. We applied the same principle, with greater resolution, to the study of SMA within Amish and Mennonite populations.

As expected, genotypes with 2 copies of *SMN2* (A1/A1, M1a/M1a, M1a/M1b, and M1a/M1c) were associated with earlier disease onset, more restricted motor development, and shorter survival than genotypes with 3 (M1a/M2) or 4 (M2/M2) *SMN2* copies. There was a broader developmental spectrum for these latter groups (Table 1) and among 9 patients with 3 *SMN2* copies, one even climbed stairs by 14 months of age (Fig 5). Other members of the M1a/M2 cohort, including her 3 siblings, walked between 17 and 42 months of age and never ascended stairs. In our clinical population, such variation is not explained by differences in *SMN1* deletion structure [18] or *SMN2* mRNA processing [32, 33]. Rather, disease expression in this particular child might have been attenuated by a rare allele outside the 5q13 SMA locus, as has been observed for the *PLS3* and *NCALD* genes [34–37].

It is not uncommon to observe phenotype variability among SMA patients with 3 or 4 copies of *SMN2* [12, 21]. More surprising, however, was the variable clinical course of A1/A1 versus M1/M1 homozygotes. The A1/A1 genotype segregated with particularly severe SMA, characterized by prenatal/perinatal onset (Table 1) and median survival of only 3.9 (as compared to 5.7) months (Fig 7A). Microsatellite and SNP analyses of the SMA locus yielded no explanation for this; A1 and M1a were similar with respect to *SMN1* deletion size and *SMN2* sequence (Fig 3)[31]. One could speculate that Amish and Mennonite families adhere to different norms of medical resource utilization. However, this would not explain the high prevalence of pre- and perinatal signs among A1/A1 (71%) as compared to M1/M1 (20%) infants and the striking uniformity of the A1/A1 phenotype, which seem to indicate a fundamental difference in disease biology.

An allele outside the 5q13 SMA locus might account for such a difference but would need to be relatively fixed within either Amish (deleterious) or Mennonite (protective) populations. More likely, there are structural differences between A1 and M1a that are in linkage disequilibrium with the *SMN1* deletion, have a functionally relevant impact on disease expression, and were undetected by our analyses. DNA marker data (Fig 3 and S2 Table) show these haplotypes to be similar but not identical, and we thus plan to use WES data to compare A1 and M1a at higher resolution.

### Natural history and implications for clinical trials

Conservative Anabaptists share core beliefs that influence their decisions about end-of-life care, particularly the application of invasive, costly, or ‘heroic’ measures for children who are otherwise helpless [38]. For infants with type 1 SMA, many Old Order Amish and Mennonite parents reject inpatient hospitalization and life-sustaining measures, which they tend to view as compounding their child’s suffering. Accordingly, we managed most type 1 SMA patients with a minimally invasive strategy in the home setting, and thus Fig 7A represents haplotype-specific survival in the absence of supportive technologies or inpatient care.

This is clearly depicted in Fig 6B, which shows a profound survival benefit for the minority of children (11%) who were fed by gastrostomy and used BiPAP. Fig 6D illustrates this principle in a different but striking way: Mennonite patients born more recently were likely to die younger [29], perhaps because the youngest generation of parents, fully informed with a molecular diagnosis and having witnessed the toll of SMA on other community members, were least likely to pursue life-sustaining treatment [39]. Here it is important to emphasize that Plain communities do not eschew modern medical technologies in general. Rather, they tend to view them pragmatically, with a sharp focus upon the potential of any intervention to actually reduce versus prolong suffering, and at what cost.

The advent of safe and effective antisense oligonucleotide (ASO) and adeno-associated viral (AAV)-based gene replacement therapies has shifted this calculus [40–42]. Knowledge of population genetics allows us to conduct targeted SMA carrier testing, identify couples at risk, and collect cord blood from their offspring. We can then use rapid, low-cost methods (Fig 3B) to determine *SMN1* mutation status and *SMN2* copy number of high-risk newborns within a few hours of life (Fig 4A). This provides a critical therapeutic advantage; data from ASO and AAV-based gene therapy trials consistently show better outcomes with earlier treatment [4, 40, 42, 43].

The opportunity to administer preventative therapies within a few days of life creates a pressing need to use appropriate historical control data to gauge their efficacy [4, 6, 40, 42]. A few prospective studies, carefully conducted in the modern era, have partially filled this gap [10, 30, 44]. For SMA patients with 2 copies of *SMN2*, data from the PNCR Network (n = 23) and NeuroNEXT (n = 20) projects align closely on the endpoint of median death or permanent ventilation between 8 and 10 months of age. Our data show that type 1 SMA follows a distinctly different trajectory within and between Anabaptist founder cohorts (Fig 7). This observation might extend to members of other founder lineages, who share relatively uniform genetic background and tend to experience common environmental, socioeconomic, and cultural conditions that shape disease expression. These facts give critical context to clinical trial data [22–24, 38, 45] and provide the appropriate framework for evaluating SMA rescue therapies administered to Amish and Mennonite babies within the first weeks of life.

## Methods

### Ethics and consent

The study was approved by the Penn Medicine-Lancaster General Hospital Institutional Review Board under a protocol entitled “Genetic Medicine and the Plain Communities (LGH IRB00000015; FWA00006038). Parents of all subjects consented to participate on behalf of their children and, where applicable, a separate written consent was obtained for reproduction of photographs (Fig 2). The individuals in this manuscript gave separate written informed consent (as outlined in PLOS consent form) to publish these case details.

### Molecular genetic methods

#### Microsatellite marker analysis

Four microsatellite markers from chromosome 5q13.2 were chosen due to their close proximity to *SMN1* and *SMN2* and validation in previous studies. Specific oligonucleotide primers were designed for each marker (S3 Table) using Primer3 (http://bioinfo.ut.ee/primer3/), and one primer was end-labeled with a fluorescent dye (either HEX or FAM). PCR was performed under standard conditions (see below) and amplicons were size-fractionated on an Appplied Biosystems (ABI) 3130 Genetic Analyzer. Alleles sizing was performed using GeneMapper software and GeneScan 500 ROX size standard (ThermoFisher Scientific, Waltham, MA).

#### Chromosomal microarray

A chromosomal microarray containing 740K SNPs (CytoScan HD Array; Affymetrix, San Diego, CA) was performed on several patients to assess haplotype sharing. These data were visualized using Affymetrix Chromosome Analysis Suite software (ChAS 3.1). Data from 5q13 were exported to Excel spreadsheets (Microsoft Corporation, Redmond, WA) for visual examination. Haplotype sharing was assessed using SNP data and 6 microsatellite markers (D5S629, UT889, D5S1370, GATA141B10, D5S1408, D5S1999; amplicon range 130 to 337 base pairs) chosen to be informative and physically close to *SMN1* and *SMN2* (Fig 2). Genome coordinates for each marker corresponded to the physical location of the forward primer using human genome build GRCh37. Haplotype analysis allowed us to assign a specific *SMN1/SMN2* genomic ‘signature’ to each patient (Figs 2 and 3).

#### Deletion mapping

Loci from the inverted 5q13 duplication were amplified and Sanger sequenced to detect the presence or absence of particular regions, and amplicons were chosen based on their ability to amplify products from multiple regions but also distinguish between the alternate products based on sequence variation contained within the fragment. Amplicons were first chosen to cover telomeric transcripts *SERF1A*, *SMN1*, *NAIP*, *GTF2H2*, *LOC647859*, and *LINC02197*. Targets were selected using BLAT (https://genome.ucsc.edu/) to identify sequence variation between the centromeric and telomeric copies, and further filtered to exclude high frequency (>1%) SNPs. Ten amplicons (S3 Table, amplicons A-J) were chosen to amplify between 2 and 3 separate loci, which could then be distinguished by their unique internal sequence.

Polymerase chain reaction (PCR) primers were designed using Primer3 (http://bioinfo.ut.ee/primer3/). A 25 μL reaction was prepared with 1U of Taq polymerase (New England Biolabs [NEB], Ipswich, MA), 200mM each of dNTP, 2.5μL of 10X PCR buffer (NEB), 1.6 μmol/L of primers, and 100ng of genomic DNA. An ABI Veriti thermocycler was used to amplify products under the following conditions: initial denaturation (3 minutes at 96°C), thermal cycling (30 sec at 96°C, 15 sec at 60°C, 90 sec at 72°C), and terminal incubation (10 minutes at 72°C). PCR products were sequenced with BigDye Terminator v3.1 Cycle Sequencing Protocol (ThermoFisher Scientific, Waltham, MA). Extension products were size-fractionated on an ABI 3130 Genetic Analyzer and analyzed using Sequencing Analysis software (ThermoFisher Scientific, Waltham, MA). Coding exons and adjacent intronic regions from *SMN2* were compared to the human reference sequence and dbSNP (http://www.ncbi.nlm.nih.gov/SNP/) to detect sequence variants.

#### SMN1/SMN2 diagnostic and copy number tests

For rapid diagnostic screening, our laboratory utilized the PCR-based procedure detailed by Dobrowolski et al., 2012 (PMID 22490618) and performed on a LightScanner 32 system (Idaho Technologies, Salt Lake City, UT).

For *SMN1* carrier testing and *SMN2* copy number analysis, competitive PCR with limiting deoxynucleotide triphosphates was used similar to the procedure outlined in Zhou et al [46]. This procedure was modified to use unique primer sets for *SMN1* and *SMN2* (S3 Table) and a different reference gene, *ALB*. An additional modification included labeling the forward primer for each amplicon with FAM to permit detection on an ABI 3130 Genetic Analyzer. Briefly, a 25 μL multiplex PCR reaction was performed using an ABI Veriti thermocycler with limiting deoxynucleotide triphosphates (6.25 μmol/L each), 1U of Taq polymerase (NEB), 2.5 μL of 10X PCR buffer (NEB), and 100 ng of genomic DNA. Primers for the target and reference gene were supplied in 3:1 ratio with a total primer concentration of 1.6 μmol/L. PCR cycling conditions were as described above.

Target and reference gene amplicons were size fractionated on an ABI 3130 Genetic Analyzer using onboard fragment analysis protocols and GeneScan 500 ROX size standard (ThermoFisher Scientific, Waltham, MA). Sample runs were analyzed using GeneMapper software (ThermoFisher Scientific, Waltham, MA). *SMN1* and *SMN2* copy number was assessed by calculating a ratio of the area under the amplicon peaks (e.g. *SMN2*/*ALB*) and comparing these to ratios of known copy number samples run in parallel.

### Patients and clinical methods

We studied 56 SMA patients born between 1965 and 2017 (175 aggregate patient-years) who lived in Canada, Pennsylvania, Ohio, Indiana, Wisconsin, Missouri, Colorado, and New York (Fig 1). Forty-two (75%) derived from Old Order Mennonite communities of the Northeastern United States, had homozygous deletions of *SMN1*, and traced to a common ancestor across 11 generations (Fig 1A). Fourteen Old Order Amish patients shared *SMN1* deletions that traced back several generations (Fig 1B).

We used available medical records and structured interviews to collect retrospective information about birth weight, growth, clinical signs, motor development, morbidity, and survival. Interviews were conducted systematically by two investigators and, in most cases, vetted using medical charts and household records.

#### Statistics

We used Prism7 software (GraphPad, La Jolla) for statistical tests. Continuous variables between two groups (e.g. A1/A1 and M1a/M1a) were tested by unpaired two-way Student’s t-test. Pearson coefficients and simple linear regression were used to test correlations between independent variables. Survival curves were analyzed using a nonparametric log-rank (Mantel-Cox) test.

## Supporting information

S1 Table Spinal muscular atrophy haplotypes. Chromosome 5q13 microsatellite marker data are listed for 42 Mennonite (M) and 14 Amish (A) patients, sorted by *SMN2* copy number (CN).
(DOCX)

S2 Table Chromosome 5q13 locus genetic markers. Representative single nucleotide polymorphism (SNP) genotypes are shown for spinal muscular atrophy haplotypes M1a/M1a, M2/M2, and A1/A1.
(DOCX)

S3 Table Diagnostic primer sets. Unique forward (F) and reverse (R) DNA primers were used for rapid and accurate detection of the *SMN1* deletion and *SMN2* copy number.
(DOCX)

S1 Fig Haplotype analysis in families lacking DNA samples from probands. For several families, one or more children carried a clinical diagnosis of SMA, but our laboratory lacked a DNA sample on the proband to confirm the diagnosis and ascertain *SMN2* copy number. In these cases, we collected samples on the parents and surviving children to establish the diagnosis through haplotype analysis. (A) Segregation of *SMN1* deletion-bearing chromosomes in a Mennonite family with 3 deceased SMA children. The father harbors the major Mennonite haplotype (M1a, shaded) and the mother carries either a doubly recombinant M1a (lacking common alleles at UT889 and D5S1408) or a novel haplotype (M1c). (B) Segregation of *SMN1* deletion-bearing chromosomes in a Mennonite family with 2 deceased SMA children. Both parents are carriers of the major (M1a) haplotype. (C-D) Segregation of *SMN1* deletion-bearing chromosomes in an extended Amish kindred with 9 deceased SMA children. All parents harbor the same *SMN1* deletion-bearing haplotype identified in two affected Amish boys.
(PDF)
