# Supplementary material for: Spinal muscular atrophy within Amish and Mennonite populations: Ancestral haplotypes and natural history
Source: PLoS One. 2018 Sep 6;13(9):e0202104. doi: 10.1371/journal.pone.0202104 (PMC6126807; doi:10.1371/journal.pone.0202104)
Supplement: S1 Table — Chromosome 5q13 microsatellite marker data are listed for 42 Mennonite (M) and 14 Amish (A) patients, sorted by SMN2 copy number (CN). (DOCX) [file pone.0202104.s001.docx]

| **Sample** | ***SMN2* CN** | **Type** | **Population** | **UT889** | **D5S1370** | **GATA141B10** | **D5S1408** |
| --- | --- | --- | --- | --- | --- | --- | --- |
| **3902** | 2 | I | M | 203/203 | 128/128 | 294/294 | 211/211 |
| **4352** | 2 | I | M | 203/203 | 128/128 | 294/294 | 211/211 |
| **5952** | 2 | I | M | 203/203 | 128/128 | 294/294 | 211/211 |
| **6832** | 2 | I | M | 203/203 | 128/128 | 294/294 | 211/211 |
| **7837** | 2 | I | M | 203/203 | 128/128 | 294/294 | 211/211 |
| **10967** | 2 | I | M | 203/203 | 128/128 | 294/294 | 211/211 |
| **11545** | 2 | I | M | 203/203 | 128/128 | 294/294 | 211/211 |
| **11710** | 2 | I | M | 203/203 | 128/128 | 294/294 | 211/211 |
| **17822** | 2 | I | M | 203/203 | 128/128 | 294/294 | 211/211 |
| **18210** | 2 | I | M | 203/203 | 128/128 | 294/294 | 211/211 |
| **19368** | 2 | I | M | 203/203 | 128/128 | 294/294 | 211/211 |
| **21048** | 2 | I | M | 203/203 | 128/128 | 294/294 | 211/211 |
| **21105** | 2 | I | M | 203/203 | 128/128 | 294/294 | 211/211 |
| **21358** | 2 | I | M | 199/203 | 126/128 | 294/294 | 211/211 |
| **23792** | 2 | I | M | 203/203 | 128/128 | 294/294 | 211/211 |
| **24352** | 2 | I | M | 203/203 | 128/128 | 294/294 | 211/211 |
| **24490** | 2 | I | M | 203/203 | 128/128 | 294/294 | 211/211 |
| **25109** | 2 | I | M | 203/203 | 128/128 | 294/294 | 211/211 |
| **26086** | 2 | I | M | 203/203 | 128/128 | 294/294 | 211/211 |
| **26703** | 2 | I | M | 203/203 | 128/128 | 294/294 | 211/211 |
| **26791** | 2 | I | M | 203/203 | 128/128 | 294/294 | 211/211 |
| **27777** | 2 | I | M | 203/203 | 128/128 | 294/294 | 211/211 |
| **33097** | 2 | I | M | 203/203 | 128/128 | 294/294 | 211/211 |
| **34673** | 2 | I | M | 203/203 | 128/128 | 294/294 | 211/211 |
| **36465** | 2 | I | A | 203/203 | 128/128 | 298/298 | 213/213 |
| **42594** | 2 | I | M | 203/203 | 128/128 | 294/294 | 211/211 |
| **44524** | 2 | I | A | 203/203 | 128/128 | 298/298 | 213/213 |
| **3971** | 3 | II | M | 203/319 | 116/128 | 294/294 | 209/211 |
| **8198** | 3 | II | M | 203/319 | 116/128 | 294/294 | 209/211 |
| **22313** | 3 | II | M | 203/319 | 116/128 | 294/294 | 209/211 |
| **26090** | 3 | II | M | 203/319 | 116/128 | 294/294 | 209/211 |
| **28647** | 3 | II | M | 203/319 | 116/128 | 294/294 | 209/211 |
| **34556** | 3 | II | M | 203/319 | 116/128 | 294/294 | 209/211 |
| **34674** | 3 | II | M | 203/319 | 116/128 | 294/294 | 209/211 |
| **40655** | 3 | II | M | 203/319 | 116/128 | 294/294 | 209/211 |
| **36737** | 4 | III | M | 319/319 | 116/116 | 294/294 | 209/209 |
| **36738** | 4 | III | M | 319/319 | 116/116 | 294/294 | 209/209 |
| **41311** | 4 | III | M | 319/319 | 116/116 | 294/294 | 209/209 |
