# Supplementary material for: Spinal muscular atrophy within Amish and Mennonite populations: Ancestral haplotypes and natural history
Source: PLoS One. 2018 Sep 6;13(9):e0202104. doi: 10.1371/journal.pone.0202104 (PMC6126807; doi:10.1371/journal.pone.0202104)
Supplement: S2 Table — Representative single nucleotide polymorphism (SNP) genotypes are shown for spinal muscular atrophy haplotypes M1a/M1a, M2/M2, and A1/A1. (DOCX) [file pone.0202104.s002.docx]

| **Chr** | **Pos** | **dbSNP ID** | **Ref** | **Het** | **M1a/M1a** | **M2/M2** | **A1/A1** |
| --- | --- | --- | --- | --- | --- | --- | --- |
| 5 | 68,351,229 | **UT889** |  |  | **203/203** | **319/319** | **203/203** |
| 5 | 68,366,143 | rs2130758 | C | 0.38 | **BB** | **AA** | **BB** |
| 5 | 68,366,875 | rs10037187 | T | 0.36 | **BB** | **BB** | **BB** |
| 5 | 68,377,251 | rs351444 | G | 0.50 | **BB** | **AA** | **BB** |
| 5 | 68,379,836 | rs163192 | C | 0.50 | **BB** | **BB** | **BB** |
| 5 | 68,387,645 | rs163194 | T | 0.50 | **BB** | **BB** | **BB** |
| 5 | 68,399,604 | rs338668 | G | 0.50 | **AA** | **AA** | **AA** |
| 5 | 68,399,722 | rs16898496 | C | 0.11 | **AA** | **AA** | **AA** |
| 5 | 68,402,578 | rs35884229 | A | 0.39 | **AA** | **BB** | **AA** |
| 5 | 68,405,526 | rs164712 | C | 0.48 | **BB** | **BB** | **BB** |
| 5 | 68,409,094 | rs164393 | A | 0.44 | **BB** | **AA** | **BB** |
| 5 | 68,409,614 | rs164392 | A | 0.31 | **AA** | **AA** | **AA** |
| 5 | 68,411,010 | rs240809 | C | 0.48 | **BB** | **BB** | **BB** |
| 5 | 68,426,312 | rs16898576 | G | 0.09 | **AA** | **AA** | **AA** |
| 5 | 68,426,570 | rs454828 | C | 0.50 | **AA** | **AA** | **BB** |
| 5 | 68,440,883 | rs337254 | C | 0.35 | **BB** | **BB** | **BB** |
| 5 | 68,448,718 | rs6887755 | G | 0.17 | **BB** | **BB** | **BB** |
| 5 | 68,449,239 | rs2932767 | G | 0.45 | **BB** | **AA** | **BB** |
| 5 | 68,449,804 | rs59776629 | G | 0.18 | **BB** | **BB** | **BB** |
| 5 | 68,471,247 | rs1128761 | G | 0.23 | **AA** | **AA** | **AA** |
| 5 | 68,488,174 | rs100192 | G | 0.50 | **BB** | **AA** | **BB** |
| 5 | 68,491,410 | rs17271763 | G | 0.22 | **AA** | **AA** | **AA** |
| 5 | 68,506,323 | rs17271888 | G | 0.07 | **BB** | **BB** | **BB** |
| 5 | 68,527,357 | rs41392244 | A | 0.22 | **BB** | **BB** | **BB** |
| 5 | 68,534,781 | rs7733529 | A | 0.34 | **AA** | **AA** | **AA** |
| 5 | 68,564,085 | rs80351595 | G | 0.12 | **BB** | **BB** | **BB** |
| 5 | 68,567,951 | rs4362903 | T | 0.46 | **BB** | **AA** | **BB** |
| 5 | 68,567,975 | rs4423955 | C | 0.41 | **BB** | **AA** | **BB** |
| 5 | 68,568,858 | rs34584424 | C | 0.03 | **AA** | **AA** | **AA** |
| 5 | 68,569,259 | rs12656423 | G | 0.32 | **AA** | **AA** | **AA** |
| 5 | 68,572,668 | rs4077460 | C | 0.46 | **AA** | **BB** | **AA** |
| 5 | 68,576,014 | rs12656918 | C | 0.27 | **AA** | **AA** | **AA** |
| 5 | 68,581,389 | rs3890705 | G | 0.40 | **AA** | **BB** | **AA** |
| 5 | 68,585,070 | rs72767873 | C | 0.15 | **AA** | **AA** | **AA** |
| 5 | 68,585,137 | rs7720249 | A | 0.12 | **AA** | **AA** | **AA** |
| 5 | 68,587,900 | rs6866993 | A | 0.43 | **BB** | **AA** | **BB** |
| 5 | 68,588,375 | rs76434936 |  |  | **BB** | **BB** | **BB** |
| 5 | 68,597,061 | rs11952620 | C | 0.24 | **AA** | **AA** | **AA** |
| 5 | 68,602,746 | rs6881767 | G | 0.50 | **AA** | **BB** | **AA** |
| 5 | 68,607,139 | rs77690023 | G | 0.24 | **BB** | **BB** | **BB** |
| 5 | 68,638,941 | rs6879078 | T | 0.44 | **BB** | **AA** | **BB** |
| 5 | 68,646,354 | rs4976195 | C | 0.16 | **BB** | **BB** | **BB** |
| 5 | 68,670,101 | rs3797091 | A | 0.50 | **AA** | **BB** | **AA** |
| 5 | 68,685,447 | rs299085 | C | 0.48 | **AA** | **BB** | **AA** |
| 5 | 68,724,918 | rs6450041 | A | 0.34 | **BB** | **BB** | **BB** |
| 5 | 68,725,130 | rs2561159 | T | 0.50 | **AA** | **BB** | **AA** |
| 5 | 68,733,645 | rs2561182 | G | 0.50 | **AA** | **BB** | **AA** |
| 5 | 68,733,698 | rs2561183 | C | 0.50 | **AA** | **BB** | **AA** |
| 5 | 68,740,653 | rs11955686 | A | 0.30 | **AA** | **AA** | **AA** |
| 5 | 68,745,122 | **D5S1370** |  |  | **129/129** | **116/116** | **129/129** |
| 5 | 68,798,118 | rs34221525 | G | 0.11 | **BB** | **BB** | **BB** |
| 5 | 68,804,744 | rs73116901 | T | 0.17 | **BB** | **BB** | **BB** |
| 5 | 68,824,670 | rs78039662 | A | 0.03 | **AA** | **AA** | **AA** |
| 5 | 68,826,246 | rs79372623 |  |  | **AA** | **AA** | **AA** |
| 5 | 69,345,350 | **SMN2** |  |  | **2** | **4** | **2** |
| 5 | 70,220,768 | **SMN1** |  |  | **0** | **0** | **0** |
| 5 | 70,671,939 | rs4704164 | C | 0.30 | **BB** | **BB** | **BB** |
| 5 | 70,680,128 | rs6453402 | C | 0.22 | **AA** | **AA** | **AA** |
| 5 | 70,680,532 | rs6453403 | A | 0.43 | **BB** | **BB** | **BB** |
| 5 | 70,688,402 | rs114748407 |  |  | **BB** | **BB** | **BB** |
| 5 | 70,690,421 | rs6884443 | T | 0.07 | **AA** | **AA** | **AA** |
| 5 | 70,704,077 | rs6453529 | G | 0.47 | **AA** | **AA** | **AA** |
| 5 | 70,710,447 | rs7727576 | G | 0.35 | **BB** | **BB** | **BB** |
| 5 | 70,716,555 | rs7443752 | A | 0.45 | **AA** | **AA** | **AA** |
| 5 | 70,717,078 | rs112612200 | G | 0.04 | **BB** | **BB** | **BB** |
| 5 | 70,719,364 | **GATA141B10** |  |  | **294/294** | **294/294** | **298/298** |
| 5 | 70,738,250 | rs55954005 | C |  | **BB** | **BB** | **BB** |
| 5 | 70,746,884 | **D5S1408** |  |  | **211/211** | **209/209** | **213/213** |
| 5 | 70,752,886 | rs115250997 | T | 0.02 | **BB** | **BB** | **BB** |
| 5 | 70,753,934 | rs7448990 | G | 0.50 | **AA** | **AA** | **AA** |
| 5 | 70,759,757 | rs76232631 | C | 0.03 | **AA** | **AA** | **AA** |
| 5 | 70,780,220 | rs10942533 | C | 0.29 | **AA** | **AA** | **AA** |
| 5 | 70,780,589 | rs6452796 | C | 0.19 | **BB** | **BB** | **BB** |
| 5 | 70,780,630 | rs3890749 | C | 0.48 | **BB** | **BB** | **BB** |
| 5 | 70,781,418 | rs4337836 | T | 0.49 | **AA** | **AA** | **AA** |
| 5 | 70,786,213 | rs115176590 | A | 0.03 | **AA** | **AA** | **AA** |
| 5 | 70,792,199 | rs16876019 | C | 0.04 | **BB** | **BB** | **BB** |
| 5 | 70,809,169 | rs1698063 | A | 0.49 | **BB** | **BB** | **BB** |
| 5 | 70,818,150 | rs12187098 | C | 0.04 | **AA** | **AA** | **AA** |
| 5 | 70,831,641 | rs156756 | C | 0.44 | **BB** | **BB** | **BB** |
| 5 | 70,832,031 | rs76186252 | A | 0.03 | **AA** | **AA** | **AA** |
| 5 | 70,833,340 | rs276593 | T | 0.50 | **AA** | **AA** | **AA** |
| 5 | 70,833,512 | rs156755 | T | 0.49 | **BB** | **BB** | **BB** |
| 5 | 70,834,926 | rs276589 | T | 0.46 | **BB** | **BB** | **BB** |
| 5 | 70,840,233 | rs182190 | C | 0.49 | **BB** | **BB** | **BB** |
| 5 | 70,842,076 | rs10942683 | C | 0.46 | **AA** | **AA** | **AA** |
| 5 | 70,856,378 | rs277939 | A | 0.48 | **AA** | **AA** | **AA** |
| 5 | 70,857,393 | rs6872235 | G | 0.29 | **BB** | **BB** | **BB** |
| 5 | 70,858,194 | rs277941 | C | 0.46 | **AA** | **AA** | **AA** |
| 5 | 70,858,268 | rs17276250 | A | 0.04 | **AA** | **AA** | **AA** |
| 5 | 70,876,592 | rs277963 | T | 0.49 | **BB** | **BB** | **BB** |
| 5 | 70,885,538 | rs465073 | T | 0.49 | **AA** | **AA** | **AA** |
| 5 | 70,907,495 | rs6885523 | G | 0.47 | **BB** | **BB** | **BB** |
| 5 | 70,907,538 | rs464182 | G | 0.47 | **AA** | **AA** | **AA** |
| 5 | 70,908,492 | rs4703617 | C | 0.49 | **BB** | **BB** | **BB** |
| 5 | 70,917,945 | rs277987 | C | 0.47 | **AA** | **AA** | **AA** |
| 5 | 70,921,768 | rs277993 | G | 0.46 | **AA** | **AA** | **AA** |
| 5 | 70,922,222 | rs16903487 | C | 0.03 | **BB** | **BB** | **BB** |
| 5 | 70,922,616 | rs12516456 | G | 0.28 | **AA** | **AA** | **AA** |
| 5 | 70,922,968 | rs277996 | G | 0.49 | **AA** | **AA** | **AA** |
| 5 | 70,923,358 | rs282357 | C | 0.47 | **BB** | **BB** | **BB** |
| 5 | 70,927,686 | rs277979 | C | 0.46 | **BB** | **BB** | **BB** |
| 5 | 70,927,923 | rs6861176 |  |  | **BB** | **BB** | **BB** |
| 5 | 70,941,094 | rs16869487 | G | 0.19 | **AA** | **AA** | **AA** |
| 5 | 70,959,669 | rs73124712 | C | 0.28 | **AA** | **AA** | **AA** |
| 5 | 70,966,060 | rs277920 | C | 0.50 | **AA** | **AA** | **BB** |
| 5 | 70,966,448 | rs277919 | G | 0.43 | **BB** | **BB** | **AA** |
| 5 | 70,966,713 | rs6867737 | C | 0.10 | **BB** | **BB** | **BB** |
| 5 | 70,967,163 | rs6872371 | C | 0.14 | **BB** | **BB** | **BB** |
| 5 | 70,967,633 | rs621292 | C | 0.31 | **BB** | **BB** | **AA** |
