# Supplementary material for: Spinal muscular atrophy within Amish and Mennonite populations: Ancestral haplotypes and natural history
Source: PLoS One. 2018 Sep 6;13(9):e0202104. doi: 10.1371/journal.pone.0202104 (PMC6126807; doi:10.1371/journal.pone.0202104)
Supplement: S3 Table — Unique forward (F) and reverse (R) DNA primers were used for rapid and accurate detection of the SMN1 deletion and SMN2 copy number. (DOCX) [file pone.0202104.s003.docx]

| **AMPLICON** | **F PRIMER** | **R PRIMER** | **SIZE (bp)** | **COORDINATES (GRCh37)** | **COORDINATES 2** | **COORDINATES 3** |
| --- | --- | --- | --- | --- | --- | --- |
| **D5S1370** | GGCTTCATGAGCCATATTTGA | GAAAAGGGGACGAAATGGTTAT | 130 | chr5:68,745,122-68,745,251 |  |  |
| **D5S1408** | TTCTGTGGCTCATGTAGCGTA | TCTCCTGCCTCACTCATCCT | 337 | chr5:70,746,884-70,747,220 |  |  |
| **GATA141B10** | GGTGATGACCACTGGCTTATACTT | TGGGAAAAAAAGATGGATAGACG | 294 | chr5:70,719,364-70,719,657 |  |  |
| **UT889** | AAGATCTCAGCATTGCACTGAA | TAATTCCAGCTGTGGGGTTCT | 209 | chr5:68,351,229-68,351,437 |  |  |
| **ALB** | TCTCCAGTAAACTGAAGGAATGCT | TCCTTTGCCTCAGCATAGTTTT | 160 | chr4:74,279,177-74,279,336 |  |  |
| **SMN1_CN** | TCCTTTATTTTCCTTACAGGGTTTC | CATTTGTTTTCCACAAACCATAAA | 142 | chr5:70,247,749-70,247,890 |  |  |
| **SMN2_CN** | TCCTTTATTTTCCTTACAGGGTTTT | CATTTGTTTTCCACAAACCATAAA | 130 | chr5:69,372,329-69,372,470 |  |  |
| **SMN_DX** | TTCCTTTATTTTCCTTACAGGGTTT | ACCTTCCTTCTTTTTGATTTTGTCT | 51 | chr5:70,247,748-70,247,798 | chr5:69,372,328-69,372,378 |  |
| **A** | AGGCAATTTCATCCTTGAGTGA | GGCACAATCTCCACTTACAACA | 459 | chr5:70,387,628-70,388,086 | chr5:68,831,445-68,831,903 |  |
| **B** | GACGGCATGTTCTCATTTACG | CTTGGCTCATTGCAGCTTCT | 509 | chr5:71,064,109-71,064,617 | chr5:70,418,427-70,418,935 | chr5:69,563,281-69,563,789 |
| **C** | ACTTGGTCTTGGTTCCTGACAC | GAGCACAGGACCTTTTGTCTTC | 374 | chr5:71,024,670-71,025,043 | chr5:70,457,975-70,458,348 | chr5:69,602,831-69,603,204 |
| **D** | AGCTGCCTAATGTCACACACTTT | AGGTTTTCCAAATAGCCAGTCA | 420 | chr5:70,907,409-70,907,828 | chr5:70,031,990-70,032,409 |  |
| **E** | TGGTTTCTTTTGGCAGTTTACC | TTTGCCATGTTGGTCAGGTT | 491 | chr5:70,218,086-70,218,576 | chr5:69,342,679-69,343,169 |  |
| **F** | ACTCCTGACCTTGTGATCTGCT | CATGAAACTGCCATGAGTATTGA | 483 | chr5:70,255,692-70,256,174 | chr5:69,380,238-69,380,720 |  |
| **G** | TTGTAAAGGCACCTACTTGCTAAAA | TCATCTAACTCCCACTCAACAGC | 398 | chr5:70,256,109-70,256,506 | chr5:69,380,655-69,381,052 |  |
| **H** | TTACAGGCATGCACCACCAT | CACATCTGAGCACAGGCTGTTA | 461 | chr5:70,259,848-70,260,308 | chr5:70,392,627-70,393,086 | chr5:69,384,401-69,384,861 |
| **I** | CAGAAAAACTGAGATTGCTCCTAGA | CACTGATGTACAAAAAGTGCGTAAA | 446 | chr5:70,679,891-70,680,336 | chr5:22,150,739-22,151,184 |  |
| **J** | CCATGGCAGTCTTGTGAGTTTG | GAAATTAGGCTGGGCGCTGT | 475 | chr5:70,330,613-70,331,087 | chr5:69,743,597-69,744,071 | chr5:68,888,442-68,888,916 |
| **SMN1/2 exon 1** | ACAAATGTGGGAGGGCGATAAC | GCCTCCACTCAACGCTATCAAG | 461 | chr5:70,220,770-70,221,230 | chr5:69,345,352-69,345,812 |  |
| **SMN1/2 exon 2** | TGGAGTAAAGTCACATAACCTCTAACC | AGGCTATCAACTTCTAAAGGAGGATATC | 425 | chr5:70,234,486-70,234,910 | chr5:69,359,062-69,359,486 |  |
| **SMN1/2 exon 3** | GGGTGGTCAAAAGAGAAAATAGGTG | TGGAACACATGTCCTGATTTTAGCA | 491 | chr5:70,237,077-70,237,567 | chr5:69,361,653-69,362,143 |  |
| **SMN1/2 exon 4** | AGACCCCAAGCCTTTCTCATTAC | AACAGTTTCTCATCTAGTCTCTGCTT | 407 | chr5:70,238,040-70,238,446 | chr5:69,362,616-69,363,022 |  |
| **SMN1/2 exon 5** | TCTGTCCGATCTACTTTCCCCA | GCTACAAAAGTTTCATGGGAGAGC | 474 | chr5:70,238,322-70,238,795 | chr5:69,362,898-69,363,371 |  |
| **SMN1/2 exon 6** | GTCCTCTAAGTTGGCATGTATAGCT | ACCCATTAGAATCTGGCCCAAG | 595 | chr5:70,240,102-70,240,696 | chr5:69,364,679-69,365,273 |  |
| **SMN1/2 exon 7** | GACCTCGTCTTTGTTTAGGGGAA | GGACAATGCAAGAGTAATTTAAGCCT | 426 | chr5:70,241,738-70,242,163 | chr5:69,366,313-69,366,738 |  |
| **SMN1/2 exon 8** | GCCACTGCAAGAAAACCTTAAC | GTTTCTTCCACATAACCAACCAGT | 509 | chr5:70,247,540-70,248,048 | chr5:69,372,120-69,372,628 |  |
| **SMN1/2 exon 9** | ATTCGTCAAGCCTCTGGTTCTA | TACAATGAACAGCCATGTCCAC | 547 | chr5:70,248,229-70,248,775 | chr5:69,372,809-69,373,355 |  |
