# Supplementary figures and images for: Spinal muscular atrophy within Amish and Mennonite populations: Ancestral haplotypes and natural history
Source: PLoS One. 2018 Sep 6;13(9):e0202104. doi: 10.1371/journal.pone.0202104 (PMC6126807; doi:10.1371/journal.pone.0202104)

A

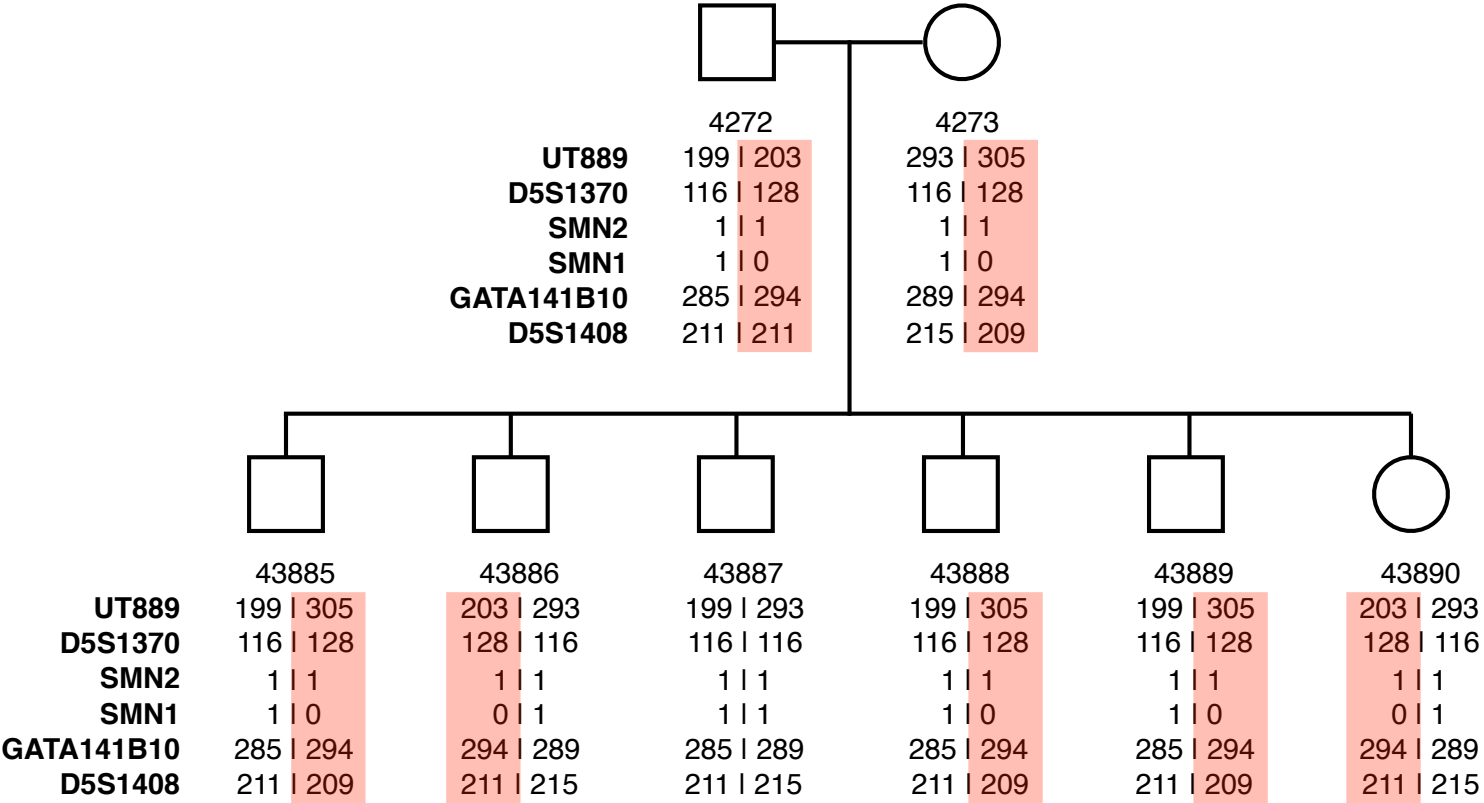

B

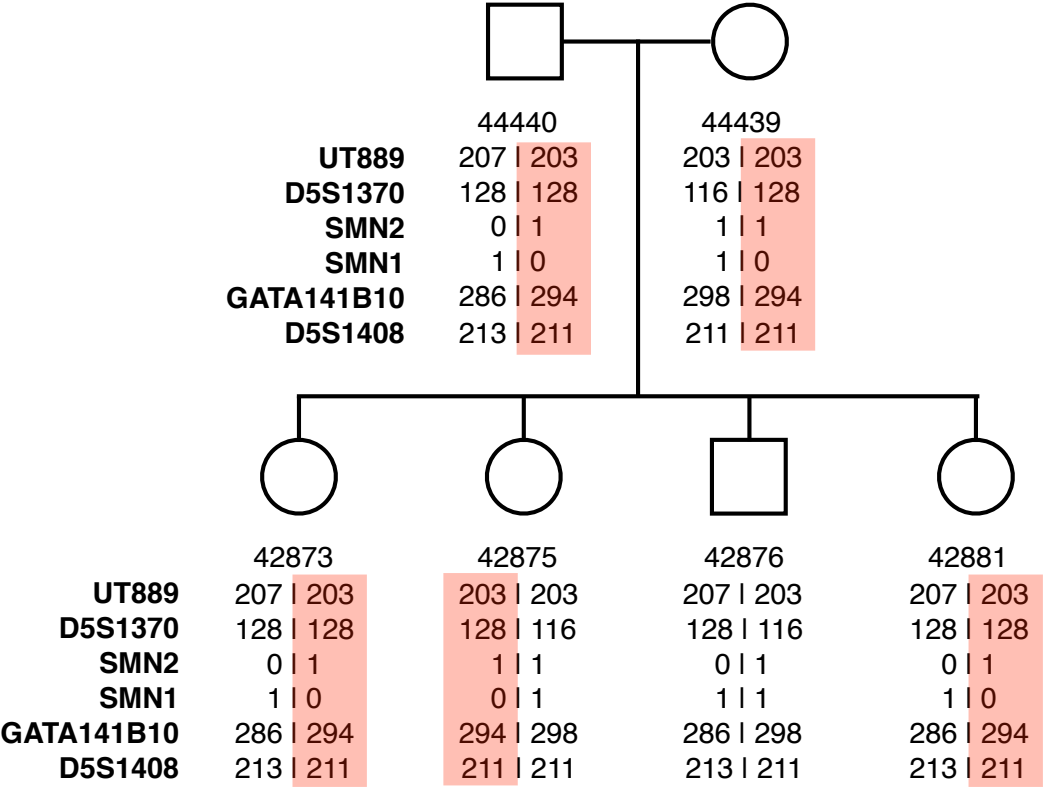

C

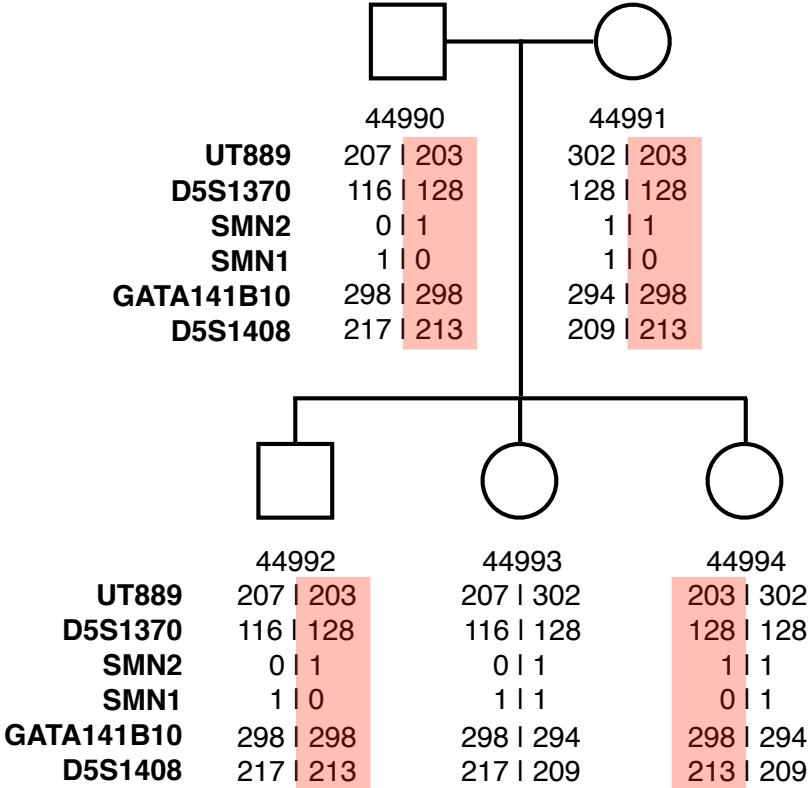

D

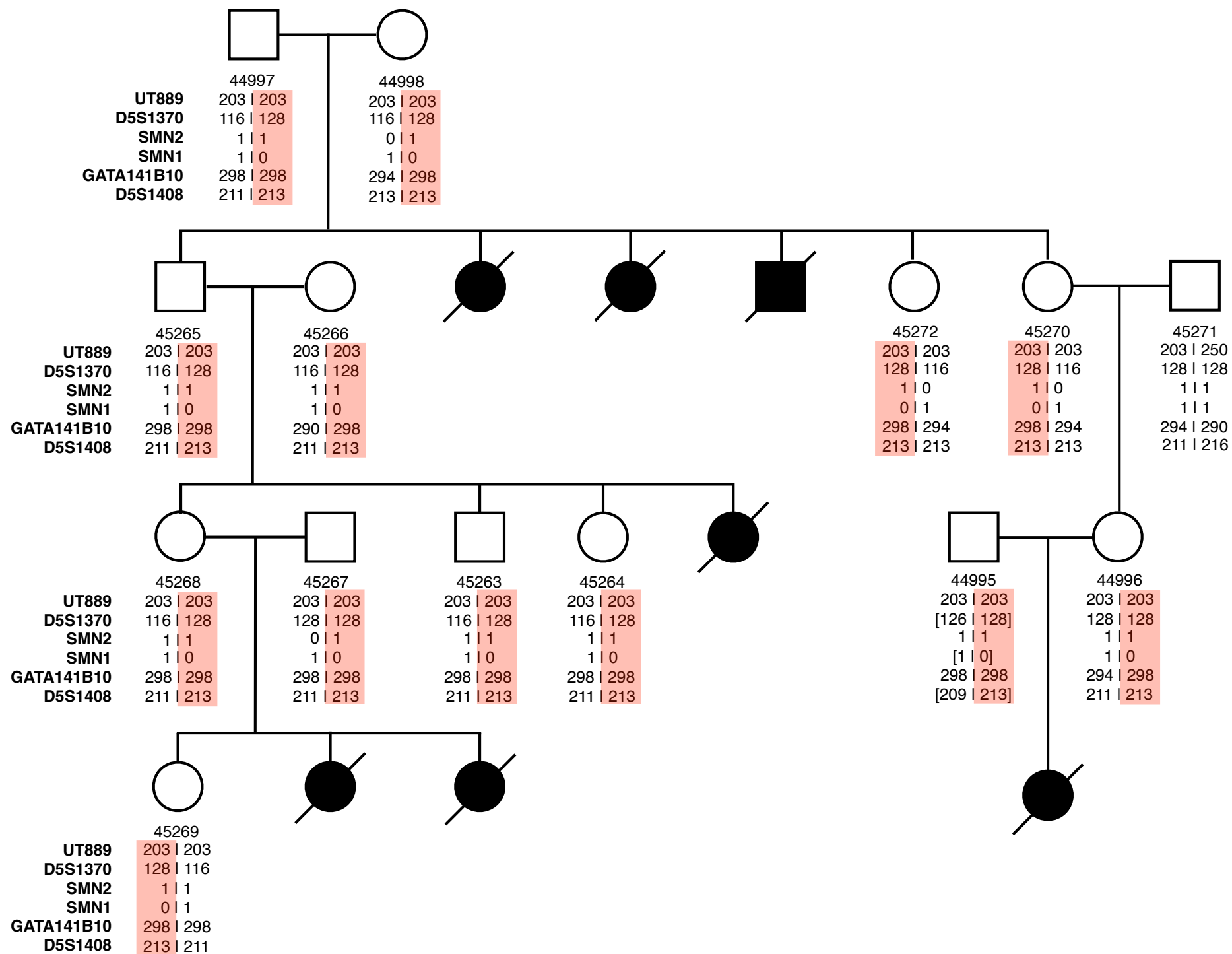

Supplement: S1 Fig — For several families, one or more children carried a clinical diagnosis of SMA, but our laboratory lacked a DNA sample on the proband to confirm the diagnosis and ascertain SMN2 copy number. In these cases, we collected samples on the parents and surviving children to establish the diagnosis through haplotype analysis. (A) Segregation of SMN1 deletion-bearing chromosomes in a Mennonite family with 3 deceased SMA children. The father harbors the major Mennonite haplotype (M1a, shaded) and the mother carries either a doubly recombinant M1a (lacking common alleles at UT889 and D5S1408) or a novel haplotype (M1c). (B) Segregation of SMN1 deletion-bearing chromosomes in a Mennonite family with 2 deceased SMA children. Both parents are carriers of the major (M1a) haplotype. (C-D) Segregation of SMN1 deletion-bearing chromosomes in an extended Amish kindred with 9 deceased SMA children. All parents harbor the same SMN1 deletion-bearing haplotype identified in two affected Amish boys. (PDF) [file pone.0202104.s004.pdf]
